# Unexplained recurrent high fever observed in a depressed adolescent

**DOI:** 10.1186/s12888-024-05705-3

**Published:** 2024-04-16

**Authors:** Xunyi Guo, Yuning Li, Lu Bai, Feng Lin, Jing Chen, Tao Zou

**Affiliations:** 1 Department of Psychiatry, The Affiliated Hospital of Guizhou Medical University, 550004 Guiyang, Guizhou Province China; 2 Department of Clinical Medicine, Guizhou Medical University, Guiyang, China

**Keywords:** Adolescent depression, High fever, Fever of unknown origin, Stress

## Abstract

**Background:**

Depressive episodes in adolescents are often accompanied by various physical symptoms, but few studies have explored the association between depression and fever, This case study is the first to report the relationship between unexplained recurrent high fever and depression.

**Case presentation:**

H is a 15 year old adolescent female currently in junior year. 2 + months ago, H gradually felt depressed after a class change. Around the time, the patient suddenly developed chills with no obvious trigger and fever. H was treated with anti-infective and anti-viral treatments all of which did not show significant improvement. No significant abnormality was seen in any of the related examinations. Considering that the patient’s anxiety, depression and somatic symptoms were obvious during the course of the disease, she was given venlafaxine hydrochloride extended-release capsule 75 mg/d; tandospirone citrate capsule 10 mg Bid; alprazolam tablets 0.4 mg qn to improve mood and sleep; supplemented with transcranial repetitive magnetic stimulation therapy 2 times/d; visible light therapy 1 time/d and psychological counseling once. Over the 6 days of treatment, the patient’s body temperature gradually returned to the normal range and her mood improved significantly.

**Conclusion:**

Depression should be considered a potential cause of unexplained recurrent fevers in adolescents, even when the temperature is significantly outside the normal range.

## Background

Depression is one of the most common mental illnesses and affects the health of billions of people worldwide. Furthermore, depression has become one of the most important health problems among adolescents. A previous study indicated that the prevalence of depression in teenagers is approximately 34% [1]. Adolescent depression is a significant risk factor for suicide [2]. Individuals who experience depression during their teenage years are more likely to develop substance abuse disorder, anxiety, and other issues in adulthood [3]. Depressive episodes in adolescents are often accompanied by various physical symptoms, but few studies have explored the association between depression and fever, and recurrent high fever during depressive episodes has not been reported. Here, we describe a case of an adolescent patient with depression who presented with high fever during the course of the illness and who experienced rapid resolution of both depressive symptoms and temperature after regular antidepressant treatment. This case report aims to increase awareness among clinicians about the role of psychological factors in patients with unexplained recurrent fever, even when the temperature is well above the normal range. We also aim to provide new directions for research on directionthe aetiology of depression in adolescents.

## Case presentation

H is a 15-year-old adolescent female currently in her junior year. Two months ago, H gradually developed depressive symptoms after a class change, including a lack of desire to interact with classmates, decreased interest in school and life, and slowness. She also experienced light-hearted thoughts and relieved her mood through self-injurious behaviours such as cutting her legs with a penknife. She also developed somatic symptoms such as dizziness, head swelling, and abdominal discomfort. Furthermore, she experienced nervousness, panic, and chest tightness before PE class. The patient’s family brought her to the local hospital 1 month ago, where she was diagnosed with a depressive episode and treated with sertraline hydrochloride. The patient discontinued the medication by herself after 2–3 days (the specific dose was not available), and her depressive symptoms did not improve. After discontinuing antidepressant medication, the patient suddenly developed chills and fever without any apparent cause. The maximum temperature was 40.5 °C. Each episode lasted approximately half an hour, and the number of episodes per day was variable. The fever was accompanied by headache, which manifested as a persistent dull pain in the whole cranial vault. The patients also experienced nausea, vomiting, vomiting with stomach contents, epigastric distention and pain, and exhaustion. Therefore, the patient was admitted to the local general hospital where the diagnosis was “Fever cause: acute upper respiratory tract infection? Gastroenteritis?” The patient received cefuroxime for 7 days to treat the infection, dexamethasone sodium phosphate for 5 days to reduce inflammation, and diclofenac sodium for 7 days to lower her body temperature. Chest CT, head CT, cerebrospinal fluid examination, and abdominal plain film radiography did not reveal any abnormalities. However, the patient did not experience any improvement in depression, high fever symptoms, or other symptoms. Additionally, significant mood swings were present before each fever. After more than 10 days, the patient was admitted to Anshun People’s Hospital. During hospitalization, tumour marker analysis, a new coronavirus nucleic acid test, blood culture and identification, blood gas analysis, and anti-Branchia pneumoniae antibody tests were performed, and no abnormalities were detected. The Chlamydia pneumoniae antibody titre was 1:40, and the *Mycoplasma pneumoniae* antibody titre was 1:80. The patients received azithromycin (7 days), ceftriaxone (7 days), oseltamivir (5 days), and piperacillin sodium tazobactam (5 days), all of which were ineffective. Paroxysmal high fever recurred, and the patient’s temperature fluctuated around 39–40 °C. The patient’s depressed mood did not improve. After one month of treatment at Anshun People’s Hospital, the patient visited our respiratory medicine department for further treatment. After admission, the patient still had symptoms of high fever, with a temperature fluctuating between 38 °C and 39.5 °C. Each fever lasted approximately half an hour before the temperature returned to the normal range, and the number of episodes was variable. There were still significant mood fluctuations prior to each fever. The respiratory medicine department considered the possibility of an infectious fever, and a viral infection could not be excluded. To clarify the diagnosis, medical tests were conducted for all possible causes of fever (Table 1). The tests showed positive results for anti-cytomegalovirus IgG, anti-EBV-IgG, anti-VCA-IgG, and anti-VCA-IgG-High antibodies. Other test results showed no significant abnormalities. The patient was treated with meropenem for the infection and with XiYanPing for the virus. The patient was administered meropenem and XiYanPing for two days, and no improvement was observed. Unexplained fever occurred after significant depression and stress, with varying frequency throughout the day. The temperature fluctuated between 38 °C and 39 °C. Considering that the patient had been diagnosed with depressive episodes in previous hospitals, the patient’s headache and abdominal discomfort could be relieved by suggestive therapy. The patient's previous medical examinations were reviewed by the respiratory department.The Chlamydia pneumoniae antibody titre was 1:40, and the Mycoplasma pneumoniae antibody titre was 1:80. However, these results can occur in normal individuals, and antibody titre of 1:160 are considered clinically significant. Therefore, Mycoplasma and Chlamydia infections were not considered at this time. Additionally, the patient tested positive for anti-cytomegalovirus IgG, anti-EBV-IgG, anti-VCA-IgG, and anti-VCA-IgG-High antibodies. These results suggest that the patient had a previous viral infection. However, since these results are also present in normal individuals, it is unlikely that the current fever was caused by a viral infection. Thus, a psychiatric consultation was requested.

**Fig. 1:**
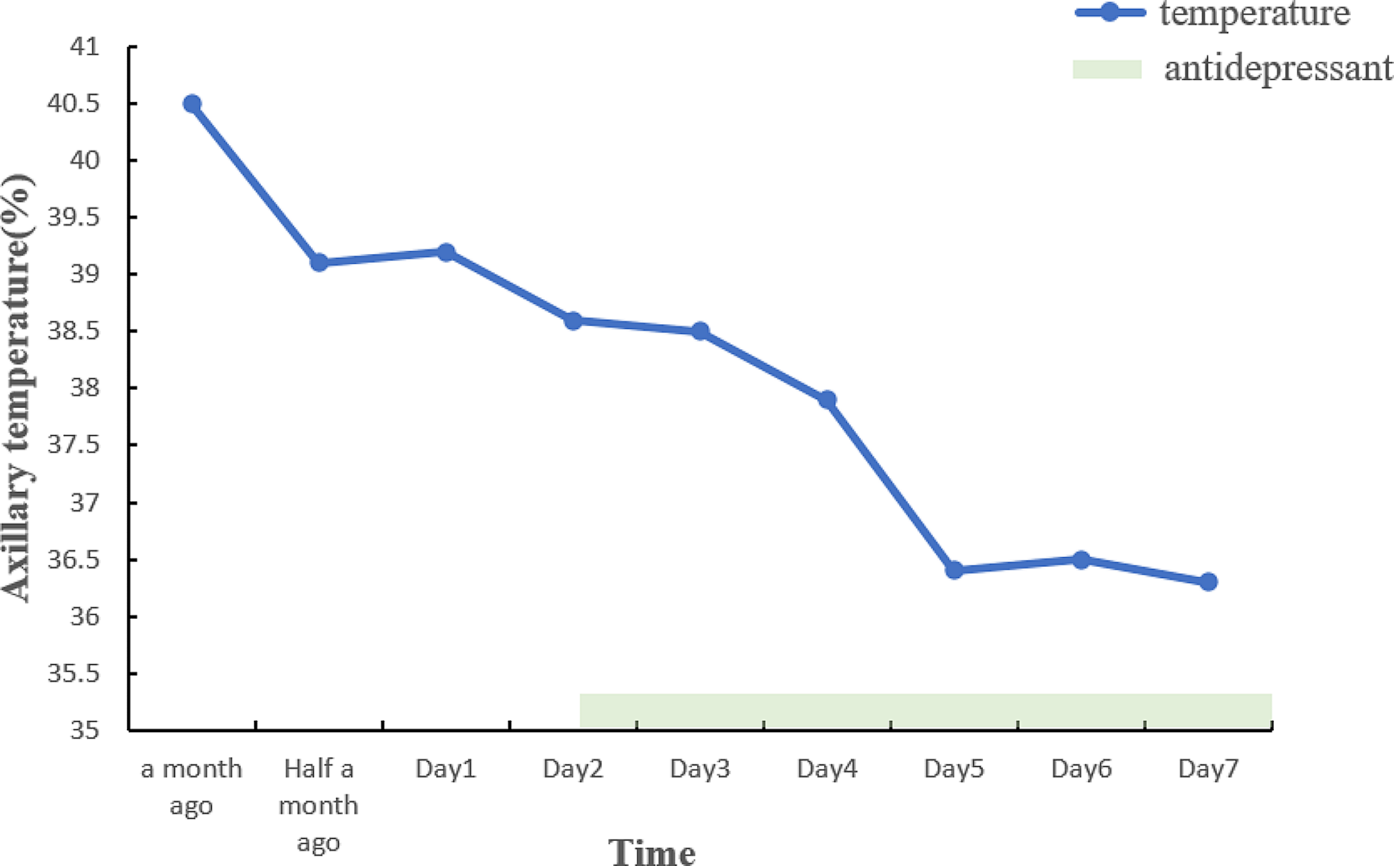
Clinical data of daily maximum temperature. *Note* The patient was transferred to our hospital after receiving treatment at other hospitals for one month. Antidepressant treatment was initiated on the second day of admission to our hospital. The patient’s mood improved after four days of treatment, while paroxysmal high fever did not recur

During the consultation, the patient had symptoms of low mood, emotional stress, reduced interest, reluctance to communicate with others, hallucinations, paranoia, etc. She had thoughts of suicide by slitting her wrists or jumping off a building, and she often relieved her mood by performing self-injurious behaviours. The patient often reported low self-esteem and guilt. The patient reported experiencing abdominal pain, headache, chills, high fever, and other physical symptoms as well as difficulty sleeping at night. She denied any prior psychiatric conditions or family history of mental illness. The patient reported good health and long-term residence in their hometown without any history of long-term residence in foreign countries or infected areas, exposure to toxic or radioactive substances, alcoholism, or smoking. The neurologic physical examination did not reveal any abnormalities. In addition, The patient exhibited febrile symptoms subsequent to experiencing depression and stress on each occasion.Overall, we believe that the patient’s current recurrent high fever, which has no clear explanation, may have bene caused by a depressive mood. We contacted the patient and her family to arrange for her transfer from the Department of Respiratory Medicine to the Department of Psychiatry. The patient’s previous use of sertraline, a 5-serotonin(5-HT) reuptake inhibitor, was less effective. Therefore, venlafaxine 75 mg/d, a noradrenergic and specific 5-HTergic antidepressant, was chosen to improve the patient’s depression. Venlafaxine was also more effective for treating somatic symptoms, pain and fever. Additionally, the patient exhibited symptoms of mood anxiety, nervousness, irritability, and poor sleep at night. To address these symptoms, tandospirone citrate (10 mg bid) and alprazolam (0.4 mg po qn) were prescribed to improve anxiety and sleep. We administered transcranial magnetic stimulation therapy twice a day and visible light therapy once a day to expedite the patient’s mood improvement, as physical therapy has been shown to have a positive impact on mood. It is important to consider that depressed mood among adolescent patients may be influenced by interpersonal and family factors. Therefore, we conducted psychological counselling based on cognitive-behavioural therapy. Over the 6 days of treatment, the patient’s body temperature gradually returned to the normal range, and her mood improved significantly(Fig. 1). The patient and her family requested to be discharged from the hospital. The patient demonstrated good adherence to treatment and consistently adhered to his medications during our several follow-up visits. Her body temperature did not fluctuate again at follow-up.

**Table 1:** Examination results for the patient

NO	Examination	Abnormal	Normal or no obvious abnormality
1	Chest CT		√
2	Cerebrospinal fluid examination		√
3	Cranial CT		√
4	Mycoplasma pneumoniae antibody + Chlamydia pneumoniae antibodies	antibody titer1:40(positive) antibody titer 1:80 (weakly positive)	
5	Mycobacterium tuberculosis antibodies		√
6	Blood culture and identification		√
7	C-reactive protein(CRP)		√
8	New coronavirus nucleic acid test		√
9	Blood cell analysis		√
10	anti-rubella virus antibodies, anti-herpes simplex virus type I/II antibodies, anti-cytomegalovirus antibodies and anti-toxoplasma antibodies	anti-cytomegalovirus antibodies IgG(positive)	
11	Lymphocyte subset assays		√
12	Immunoglobulin, single complement, Rheumatoid factor assay, Anti-streptococcal hemolysin O		√
13	Interleukin-6, Procalcitonin, Anti-cyclic citrulline antibody, Blood sedimentation		√
14	Myocardial enzyme set + Myocardial markers, Adenosine deaminase, Liver function + Kidney function + Electrolyte + Lipids		√
15	Abdominal sonography		√
16	Cardiac ultrasound		√
17	Electrocardiogram(ECG)		√
18	anti-neutrophil, anti-glomerular basement membrane, Respiratory pathogens, Typhoid antibodies, anti-nuclear antibody profile		√
19	EB virus antibody	anti-EBV-IgG, anti-VCA-IgG, and anti-VCA-IgG-High(positive)	
20	Urine routine + stool routine + stool occult blood test		√
21	Thyroid function-TSH FT3 FT4		√

## Discussion and conclusions

The patient described in this case report underwent a full course of antibiotics, antiviral agents, anti-inflammatory agents, and other regular treatments for more than a month in accordance with the principles of antibiotic use. Despite her normal examination results, the patient still experienced recurrent fever. Second, the patient reported that her fever symptoms appeared after experiencing a depressed mood, and each subsequent high fever symptom occurred in the context of dramatic mood swings and tension. Additionally, the patient discontinued antibiotics and antiviral medication prior to being transferred to the psychiatric department. The patient did not experience fever after receiving treatment with antidepressants, psychological counselling, or physiotherapy. During several follow-up visits, the patient consistently took the medication as prescribed and did not experience any further episodes of high fever. Thus, we present a case suggesting a link between recurrent unexplained symptoms of high fever and depression in adolescents. Research has demonstrated that stress can act as a stressor, projecting through the medial prefrontal cortex (mPFC) to the basolateral amygdala, the anterior paraventricular thalamus, and the accumbens nucleus shell, all of which can cause behavioural changes in depressed mice [4]. Research has demonstrated that psychosocial factors can stimulate the mPFC→hypothalamic dorsomedial nucleus pathway, resulting in an increase in body temperature in rats [5]. When the rats experienced social pressure to fail, they developed a fever with a maximum temperature of 38.5 °C [6, 7]. An association between low-grade fever depressive episodes in adolescent patients has also been found in case reports [8]. In summary, we believe that stress and depressed mood may contribute to recurrent high fever in patients. We suggest that the associations between stress and depression and recurrent fever may be mediated by the mPFC, but the exact mechanism involved remains to be investigated. It is important to remember that stress may play a significant role in the biological mechanisms of adolescent depression. This case report serves as a reminder to researchers that stress should not be overlooked when investigating the pathogenesis of depression in adolescents. Additionally, depression should be considered a potential cause of unexplained recurrent fevers in adolescents, even when the temperature is significantly outside the normal range. By doing so, patients can recover more quickly, and a harmonious doctor‒patient relationship can be promoted. This case report has several limitations, including the absence of a control group and the uniqueness of the patient’s experience.

## Data Availability

The datasets used during the current study are available from the corresponding author on reasonable request.
